# “We’re building the plane while we’re flying it”: perspectives on local cigar policy implementation from qualitative interviews with key personnel

**DOI:** 10.1186/s43058-026-00864-8

**Published:** 2026-01-16

**Authors:** Jessica King Jensen, Kathryn LaCapria, Stefanie Gratale, Myneka Macenat, Jeanne M. Ferrante, Alexandra McGarry Williams, Hassiet Asberom, Cristine D. Delnevo, Sunday Azagba

**Affiliations:** 1 Rutgers Institute for Nicotine & Tobacco Studies, New Brunswick, NJ USA; 2 Rutgers Robert Wood Johnson Medical School, Department of Family Medicine and Community Health, New Brunswick, NJ USA; 3 Rutgers School of Public Health, Piscataway, NJ USA; 4 Rutgers Cancer Institute, New Brunswick, NJ USA; 5 Rutgers Robert Wood Johnson Medical School, Department of Pediatrics, Division of Population Health, Quality, and Implementation Sciences, New Brunswick, NJ USA; 6 Rutgers Robert Wood Johnson Medical School, Piscataway, NJ USA; 7 Ross and Carol Nese College of Nursing, Pennsylvania State University, University Park, PA USA

**Keywords:** Policy implementation, Cigar, Tobacco control, Theoretical framework, Qualitative interviews

## Abstract

**Background:**

Nearly 300 US municipalities have enacted policies regulating cigar pack size and price to reduce youth access to and use of inexpensive cigars. This study characterizes the policy implementation processes of these local policies and identifies associated barriers and facilitators.

**Methods:**

Between June and November 2023, we conducted 36 semi-structured qualitative interviews with professionals involved in adopting and implementing local cigar regulations. Interview transcripts were coded and thematically analyzed using a template organizing style and iterative immersion-crystallization analysis of coded segments. Themes were categorized using the Inventory of Factors Assessing Successful Implementation and Sustainment determinant framework, encompassing domains such as external factors, internal organizational factors, retailer-specific factors, and policy-specific factors.

**Results:**

Participants described distinct education and enforcement activities post-policy adoption, often managed by separate, autonomous organizations and individuals. Key facilitators identified included state funding (external), interagency collaborations and unofficial capacity-building efforts (internal), and clear, enforceable ordinances with less retailer pushback (policy-specific). Conversely, significant barriers included state-level influences (external), lack of standardized protocols, resource disparities, and varied implementer perspectives (internal). Retailer-specific barriers included limited English proficiency and a willingness to risk violations. Policy-specific challenges involved confusing cigar definitions and insufficient deterrent penalties.

**Conclusions:**

Local cigar policy implementation often involves multiple autonomous organizations and individual implementers. The salience of identified barriers across various contexts may have important implications for policy impact. Understanding the facilitators and barriers to policy implementation may enable other localities to proactively develop strategies to increase success.

**Supplementary Information:**

The online version contains supplementary material available at 10.1186/s43058-026-00864-8.

Contributions to the literature

- Factors supporting or detracting from policy implementation are understudied, particularly with regard to broader tobacco policy implementation.
- Qualitative interviews with implementation actors provide rich data to understand how policies are practically applied in real world settings.
- Local cigar policy implementation often involves multiple autonomous organizations and individual implementers which presents barriers.
- The use of an implementation science framework to synthesize facilitators and barriers to implementation may enable localities to proactively develop processes to increase success.

## Background

Cigars represent the most prevalent combustible product used among youth in the United States (US), with over 500,000 adolescents reporting past 30-day cigar use in 2023 [1]. Despite comparable health risks to cigarettes [2], cigars remain less regulated. The tobacco industry actively exploits loopholes in existing tobacco regulations, marketing and selling mass machine-manufactured cigars in ways appealing to youth, including inexpensive small packs (e.g., one for $0.49 or five for $1.00) [3, 4] and flavored varieties. In response, nearly 300 municipalities across the US have adopted policies regulating cigar pack size and price. These policies, which increase the minimum *price* per package or minimum *number* of cigars required to be sold within a package, are primarily intended to reduce access to and use of inexpensive cigars [5].

The earliest cigar pack size and price policies were adopted in Huntington Park, California and Boston, Massachusetts in 2011. The Huntington Park policy mandated that cigars be sold in packs of at least two, effectively eliminating single cigars, while the Boston policy required that cigars be sold in packs of at least four unless sold for at least $2.50 per cigar. Today, municipalities employ various combinations of policies regulating minimum cigar prices, minimum pack sizes, or both [5]. The impact of these policies varies. Three studies assessing changes within the retail environment post-policy adoption noted increases in sale prices and decreases in single cigar availability [6–8]. Similarly, an analysis of the policies' impacts on cigar sales showed that they were associated with sales decreases [9]. However, the effects on youth and adult cigar use have been mixed, with some studies reporting decreases in use [10, 11], while others identified no significant change [10]. Differences in policy impact may result from differences in how policies are implemented in the real world. Indeed, emerging literature on the implementation of other tobacco policies suggests there may be important gaps in how policies are interpreted or enforced across municipalities. This variability in effectiveness represents a significant public health problem. It suggests that in many municipalities, these policies may be failing to achieve their primary goal of reducing cigar use and leaving youth vulnerable to a product category popular for initiation. Further, this lack of uniform success creates the potential for health disparities and indicates that the tobacco industry may be successfully circumventing policy intent through loopholes in policy language or weaknesses in enforcement. Therefore, it is essential to evaluate how specific features of cigar pack size and price policies, including their implementation processes, may contribute to policy success and may influence use patterns, to identify best practices that inform future policy adoption.

Implementation science offers well-developed frameworks for understanding how interventions are put into practice, particularly within clinical settings [12]. However, these frameworks have been used sparingly in population-based or policy contexts [13]. This study addresses this gap by assessing the real-world implementation of local cigar pack size and price policies, drawing insights from the perspectives of key personnel involved. We conducted interviews with key informants that were guided by the Stages Framework, which delineates five cyclical stages in the policy process: problem definition and agenda setting, policy formulation, policy adoption, policy implementation, and policy evaluation [14]. This qualitative analysis focused specifically on the policy implementation stage, aiming to understand how policies are practically implemented, including both facilitators and barriers to implementation.

To contextualize the identified themes within the broader framework of implementation science theory, we utilized the Inventory of Factors Assessing Successful Implementation and Sustainment (IFASIS) tool [15]. Recognizing that interventions, including policies, are implemented within complex, multilayered, and dynamic contexts, the IFASIS was designed to understand contextual factors influencing the implementation process. The IFASIS tool synthesizes common implementation science theories, including the Consolidated Framework for Implementation Research (CFIR), Exploration, Preparation, Implementation, Sustainment (EPIS), Normalization Process Theory, and the Health Equity Implementation Framework, to identify four interacting domains of influence: (1) factors about the intervention, (2) factors about people receiving the intervention, (3) factors within the organization implementing the intervention, and (4) factors outside the organization. Equity is integrated throughout the framework, highlighting the importance of its consideration across all domains. Constructs from leading implementation science theories are included within each domain.

**Fig. 1:**
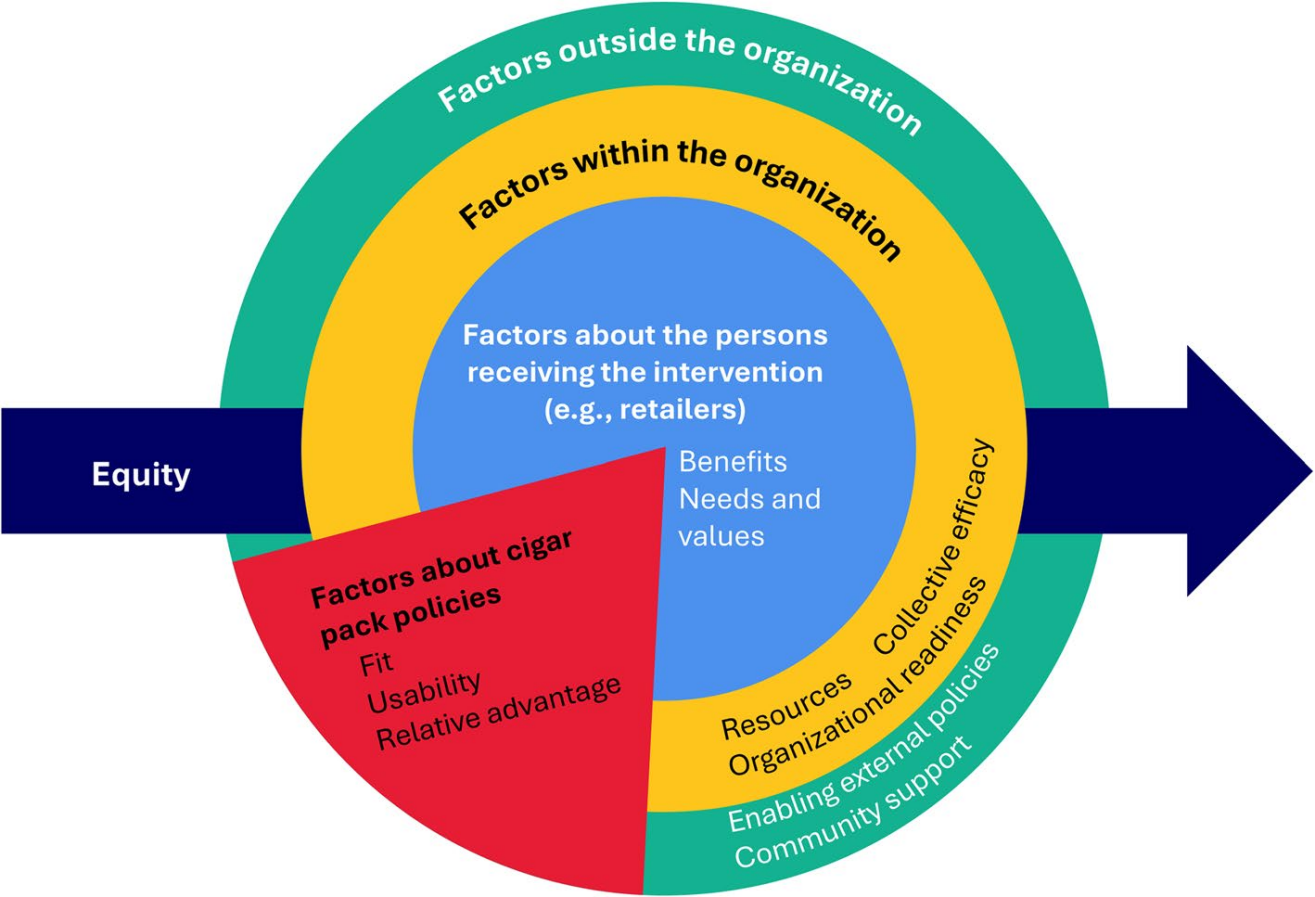
Application of the IFASIS Determinant Framework to Cigar Pack Policies as an Intervention

Figure 1 depicts the IFASIS tool as applied to cigar pack policies. In this context, the policies serve as the "intervention" with specific policy components potentially influencing implementation through their fit, usability, or relative advantage over other policies. Tobacco retailers represent the primary "population of focus" for implementation, and understanding the potential benefits they receive from the policy, along with their needs and values, may be critical for successful policy implementation. Multiple organizations with distinct characteristics and priorities may be involved in policy implementation, highlighting the importance of examining the influential "factors within these organizations," such as necessary resources or readiness to implement the policy. Lastly, "factors outside the organizations," such as public perceptions or cigar smoking rates, may significantly influence how cigar pack policies are implemented.

## Methods

Between June and November 2023, we conducted 36 semi-structured qualitative interviews with professionals involved in adopting and implementing local cigar pack regulations in California, Minnesota, Massachusetts, and New York using a content analysis approach. Participants were recruited from these states because California, Minnesota, and Massachusetts are the only three US states with multiple cigar pack policies, and New York City’s policy changed over time. Having multiple cigar pack policies throughout a state, particularly varying policies, allowed us to hear different perspectives on policy components and facilitated recruitment. Participants were purposively recruited based on their direct experience adopting, implementing, or evaluating cigar pack size policies. Initial contact lists were identified during our 2021 policy search and synthesis [5], with additional participants identified using snowball sampling. Potential participants were contacted by email. In total, 226 individuals or organizations were contacted. Of these, 89 responded, and 39 agreed to be interviewed. A total of 36 interviews were completed with 37 participants; one interview included two staff members from the same municipality. We were unable to conduct interviews with the remaining two interested participants due to scheduling conflicts. The participants' experiences ranged from less than one year in their role to over 30 years, representing local and state health departments, non-profit organizations, and technical assistance groups. The Rutgers Institutional Review Board approved the study, and the COREQ guidelines were used in reporting this work (Additional File 1).

Prior to the interviews, the study team developed a semi-structured interview guide, which was pilot tested and refined. The Stages Framework [14] formed the basis for the interview guide, with questions focused on the adoption process, the implementation process, and the effectiveness of policies (Additional File 2). Interviews were conducted on Zoom and lasted up to 60 min. Each interview included at least one moderator and one notetaker. Three researchers (two with PhD degrees and one with an MS degree), all female and trained in qualitative interviewing techniques and the study content, conducted the interviews. The research team had no prior relationships with participants. Interviews were audio-recorded and transcribed verbatim, and field notes were used to provide additional contextual information.

We conducted thematic analysis of interview transcripts using a template organizing style and immersion/crystallization analysis of coded segments [16–18]. We created a preliminary codebook to tag and sort data using concepts from the interview guide and initial readings of two transcripts. Three analysts independently coded these two transcripts to assess the clarity and relevance of the codes and definitions. The team met weekly to discuss coding uncertainties and discrepancies, which were resolved through group consensus. The codebook was revised, and coding schemes were refined accordingly. The three analysts then independently coded the remaining transcripts using Atlas.ti 22 software. Subsequently, coded data were analyzed using a series of immersion/crystallization cycles, where segments were read and interpreted until themes crystallized [18]. During weekly team meetings, we organized emerging themes into meaningful categories, identified connections between different categories, and refined, compared, and contrasted themes between participants. The final immersion/crystallization cycle focused on identifying commonalities and variations of themes related to domains from the Inventory of Factors Assessing Successful Implementation and Sustainment (IFASIS) determinant framework: factors outside the organization, factors inside the organization, factors about tobacco retailers, and factors about the policy (Fig. 1) [15]. The present study focuses exclusively on policy implementation. Facilitators and barriers are identified, and selected quotations demonstrate our key findings.

## Results

### Local policy implementation components and process

Participants described the standard policy implementation processes that occurred within their municipalities. Across all interviews, two primary activities emerged: retailer education and enforcement. For each activity, participants discussed policy implementation timelines, the utility of in-person educational visits, and various strategies employed to monitor compliance and enforce the policies. Specific implementation strategies identified through these conversations are provided in Table 1 and discussed below. Exemplar quotes for all themes are within Additional Table 3.

**Table 1:** Local Cigar Policy Implementation Strategies Identified in Qualitative Interviews

• Extending the grace period between policy adoption and enforcement to allow for product sell-down and education • Inter-municipality collaboration for strategizing and sharing best practices • Coordinating implementation efforts across all pertinent organizations • Disseminating policy provisions via formal letters to retailers • Providing education materials in multiple languages • Creating visual aids for educational purposes • Delivering in-person education to retailers • Conducting follow-up education visits • Conducting in-person monitoring/inspections • Administering penalties for violations	

#### Grace period between adoption and enforcement

Most participants reported similar timelines and sequential steps between policy adoption and enforcement. Typically, policy implementers mailed retailers a letter detailing the policy change, followed by initial in-person education, before any penalties would be administered. This "grace period," frequently referenced by participants, usually spanned from one to six months. Most participants found the grace period beneficial, as it allowed retailers time to liquidate affected products and provided tobacco control, enforcement, and other involved staff adequate time to deliver comprehensive education:

*“You'd pick an effective date for the policy to go into effect, and we would always pick a date that was usually about six weeks or more out, so that our programs could do the educational piece that they needed to do for the retailers.” (Statewide Technical Assistance Provider)*

#### In-person educational visits

Most participants described education as typically provided during in-person visits to each retailer by an individual or a team. The specific roles of those involved in the process varied by municipality, often including tobacco control staff, public health staff, or code enforcement staff. In-person visits were deemed beneficial because retailers often had limited time to check the mail for educational materials. These visits also ensured that information was directly received and understood. A Local Tobacco Control Staff member explained,

"*I visited every single store in person with a copy of that letter [describing the policy] because we do know that, you know, store owners are busy...they don't always open their mail. So it was a personal visit: 'Have you seen this letter? You need to know this, and this is what you need to do.'"* (Local Tobacco Control Staff)

Participants also highlighted the inherent complexity of some cigar pack size and price policies, particularly those with exclusions or varying requirements by cigar type. Some noted that the policies could be difficult for retailers to fully comprehend, leading to frequent questions about compliance. While not explicitly stated as the sole reason for in-person visits, the opportunity to explain intricate policy components directly to retailers appeared to be a critical strategy. Furthermore, some participants suggested that non-compliance among certain retailers might stem from insufficient policy education and a lack of understanding regarding compliance procedures:

*“In other places they weren't complying with it and they didn't really understand how to comply because they weren't very well educated on it”* (Local Tobacco Control Staff)

#### Educational materials

Participants reported using materials in multiple formats and languages to support retailer education, including signage, information sheets, postcards, flyers, and brochures with pictorials of which products could be sold and at which prices. Some of these materials were also displayed in the stores for ongoing retailer reference and customer education. These resources were consistently described as important for effective education, especially given the challenges some retailers faced in understanding some of the detailed cigar pack policies.

*“So we kind of put together educational materials, you know, with flyers and pictures of the products… when we talked about a 10-pack size, what that meant, what the exemptions to that were, because that was really confusing.”* (Local Tobacco Control Staff)

#### Monitoring compliance

Efforts to monitor policy compliance varied. Most participants mentioned that their municipalities had conducted retailer inspections to assess compliance. In this process, implementers often collaborated with a variety of partners, including environmental health divisions, police departments, departments of revenue, tobacco coalitions, and federally-funded compliance initiatives, which required ongoing coordination and trust building. The frequency of inspections was often noted as once or twice per year and was primarily driven by a municipality's available resources rather than official protocol or formal policy requirements. Other participants indicated compliance visits were often prompted by complaints from community members, primarily parents of youth who had obtained products. However, some also mentioned that retailers may call to complain about their competitors not complying with regulations. Funding for inspections was often provided through retailer licensing fees, while some participants reported receiving grant funding to address costs.

#### Enforcement actions

While enforcement actions were discussed, participants seemed to rely more heavily on corrective education over punitive measures. Typically, following education efforts, retailers found in violation would first receive a warning (official or unofficial). Repeat offenses could result in citations and penalties, with fines and permit suspension lengths increasing with each subsequent offense. Participants knew of fines and product seizures that had occurred, but these actions seemed rare. A significant barrier to consistent enforcement was that staff conducting inspections were often not authorized to directly administer citations or penalties. Instead, these actions frequently required progression through a board of health or court hearing, with retailers having options for appeal. Some participants noted that because enforcement was either a lengthy, complex process or dependent on another agency that might not prioritize tobacco policy enforcement, implementers often focused on retailer education as a more practical and immediate strategy instead.

### Facilitators and barriers to implementation

Across the aspects of the implementation process, facilitators and barriers were identified across the IFASIS domains. Table 2 summarizes the key facilitators and barriers, which are described in detail below.

**Table 2:** Factors Associated with Cigar Pack Policy Implementation Situated within the IFASIS Determinant Framework

Domain	Facilitators	Barriers
Factors related to Cigar Pack Policies	• Clear and enforceable ordinances improve implementation • Policies allow for continued sales at higher prices, resulting in relatively less retailer pushback toward cigar pack size and price policies	• Violation penalties may not be sufficient to discourage retailers from selling restricted products • Weight-based cigar definitions are challenging to implement and enforce
Factors among Retailers	None identified	• Retailers creatively evade restrictions • English proficiency among retailers is limited • Product education for retailers is insufficient • Retailers are willing to risk policy violations
Factors Within the Organization	• Implementers engage in unofficial efforts to build capacity • Implementation is a collaborative effort across responsible agencies and organizations	• Inspections and enforcement lack standard protocols • Municipalities differ in their available resources • Insufficient knowledge of the ordinance leads to partial implementation • Perceived best practices vary across implementers • Cigar use and cigar pack policies may be perceived as less of a priority than other public health and tobacco issues
Factors Outside the Organization	• Grant funding may be available to address costs related to implementation and enforcement	• States influence local implementation • Emergent events may hinder implementation efforts, boosting illegal sales • A “patchwork” of policies exists across municipalities

*IFASIS* Inventory of Factors Assessing Successful Implementation and Sustainment

#### Facilitators related to cigar pack policies

##### Clear and enforceable ordinances improve implementation

Explicit language and effective communication were mentioned as crucial elements for policy success. Interviewees emphasized the importance of ensuring a policy was enforceable *before* its adoption. Furthermore, participants noted that if adequate resources were not available for enforcement, the ordinance would likely not succeed. Specifying factors such as staffing and inspection frequency during the policy adoption stage, potentially even within the ordinance itself, was deemed necessary for proper enforcement.

##### There is less retailer pushback toward cigar pack size and price policies

Participants noted that cigar pack size and pricing policies often encountered less opposition and were relatively easier to adopt than other tobacco policy areas, such as restrictions on flavor availability. Proposed reasons for this included their integration into broader policy components (e.g., adopted alongside flavor restrictions) or the perception that the policy allowed retailers to continue selling products, potentially even for additional profit. For example,

“*Our strategy is we want to give the retailer a heads up that we're putting the price of tobacco up now; use that extra money so that you can buy a freezer or produce case and transition over to something else, because down the road we are going to ban the sale of tobacco products… use this to transition to another product that's going to allow you to stay whole as a business.”* (Local Tobacco Control Staff)

#### Facilitators within the organization

##### Implementers engage in unofficial efforts to build capacity

Participants shared that in the absence of explicit state guidance, local community departments and staff proactively collaborated with other municipalities to develop services or resources to build capacity for staff to conduct inspections and enforcement. Participants also reported informally training new staff on tobacco policies, enforcement best practices, and newly released tobacco products.

*“In some of our jurisdictions that passed tobacco retail licensing ordinances, there has not been really strong enforcement. And, so, we have developed a [redacted location] Tobacco Retail Enforcement Network to bring enforcement officials, tobacco retail enforcement officials together to try to, you know, just kind of build their capacity and their understanding about the various policies and enforcement best practices and strategies.”* (Local Tobacco Control Staff)

##### Implementation is a collaborative effort across organizations

Several different partners were often involved in the implementation process. Across jurisdictions, there were variations in who was responsible for providing signage, education, and enforcement, though in most cases, this included tobacco control staff, public health staff, code enforcement officers, or police officers. Additional departments or organizations were noted as providing information, advocacy, or resources.

#### Facilitators outside the organization

##### Grant funding may be available to address costs

Some participants noted that municipalities could apply for funding from the state to support implementation activities. Use of this funding varied, but its availability significantly improved implementation in some municipalities:

*“So [redacted] State Department of Justice has had tobacco-related enforcement grants over the past… since, like, 2018. And so there have been some local departments, local sheriff departments or police departments, that have really used that money well to kind of figure out enforcement.”* (Local Tobacco Control Staff)

#### Barriers related to cigar pack policies

##### Penalties may not be sufficient to discourage retailers from selling restricted products

Participants discussed the importance of policies having fines and penalties that are significant enough to encourage compliance, yet not so harsh that those responsible for levying fines would be reluctant to administer them. Several noted that the current structure, wherein few violations were actually administered, likely led retailers to continue selling restricted products. A participant highlighted the economic incentive:

“*These things sell, and they're trying to make money. As simple as that. I think it all boils down to the mighty dollar. And why I'm getting resistance from some of these people and doing these backdoor type of things, especially as more and more people comply. Now they're more and more and more in demand. So they're making more and more and more money, you know, so they're taking that risk. And they don't think that the penalties are all that much. They're not going to go to jail.”* (Local Public Health Staff)

##### Weight-based cigar definitions are challenging

Participants stated that weight-based definitions were confusing and often led to the misidentification of little cigars and cigarillos. Some policies utilized an antiquated federal weight-based definition, describing a cigar as weighing 3 pounds per thousand. In several instances, participants mentioned that if they had adhered to the weight-based definition, certain products would have been priced lower than intended. Furthermore, participants noted that weight-based definitions for cigars were impractical for enforcement, as inspectors would not carry scales to weigh products in stores. A Local Tobacco Control Staff member provided an example:

"*Whatever company makes Black and Mild reached out to them and said 'hey our product is not a little cigar, it exceeds, you know, the cutoff for that product. It's actually a large cigar by your ordinance definitions.' And then we've had to send out communication to all retailers and say… this is now considered a large cigar, which for them for something like Black and Mild is just preposterous to think that they're charging that much money for that. And so that then becomes another thing for them to figure out: 'Do I carry this product?’ 'How do I communicate to customers that this is now a large cigar and what was $0.99 is now $8?”* (Local Tobacco Control Staff)

#### Barriers among retailers

##### Retailers creatively evade restrictions

Participants observed that retailers found ingenious ways to sell existing non-compliant cigar packages. Examples included retailers combining two packs with tape and rubber bands to meet minimum pack size requirements, selling banned products from personal vehicles, or concealing them within the store in drawers, microwaves, and behind doors:

*“So because $2.50 is a lot to pay for a single [cigar], and you want to make that 5 pack cheaper [per stick] than the single… there were a lot of two packs around… and they taped or elasticized two two-packs. So that now it's a made-up four-pack for $5, which made it $1.25 per cigarillo.”* (Local Tobacco Control Staff)

##### English proficiency among retailers is limited

Participants noted that many retailers had limited English proficiency. Alternative methods were necessary to effectively educate them on the policy. These included providing letters in different languages, using photos of legal and illegal products, simplifying policy outlines, and employing in-person education.

##### Product education is insufficient

A few participants stressed the need for more comprehensive education resources regarding product definitions for retailers. They suggested that all parties involved in the policy adoption and implementation processes (i.e., retailers, policymakers, and staff) should be educated about which specific products fall under certain policy aspects. Participants noted that providing images of compliant and non-compliant cigars would have helped limit confusion throughout the whole process. Furthermore, participants reported that retailers often received conflicting information from their distributors.

“*I think with cigars, one of the things that we found is that retailers were really confused about what was the cigar, what was the cigarillo, because it doesn't matter if the manufacturer labeled them as cigars, if they don't meet the legal definition, they're not cigars. So it would have been helpful to have had better explanation, and I think pictures of products that they could have and they couldn't have. It was hard to explain it to them. We give them, you know, all this information, they didn't want to read it and they, even when they would read it, they didn't understand. So having real clear, I think, pictures of acceptable products and not acceptable products [would be helpful].”* (Local Tobacco Control Staff)

##### Retailers are willing to risk policy violations

Participants observed that some retailers appeared willing to risk violating policies. It was noted that during the policy adoption process some retailers expressed concern about their ability to stay in business if the policy reduced sales, which may lead some retailers to continue to offer products in smaller pack sizes. Additionally, monitoring and compliance checks were infrequent (e.g., occurred once per year or every two years), which may encourage retailers to take such risks.

#### Barriers within the organization

##### Inspections and enforcement lack protocols

Several participants reported the absence of standardized protocols or procedures for conducting inspections, ensuring safety during visits, defining enforcement actions, and administering violations. Few participants had received specific training for implementing tobacco retail policies, having instead been trained in broader areas such as health education, food safety inspections, or general law enforcement.

Participants also elaborated on the inherent difficulties in enforcing policies. While they were generally aware that fines and suspensions were stipulated in the guidelines, the practical process from an enforcement agent identifying a violation to a retailer receiving a penalty was often unclear or deemed infeasible. Participants noted difficulties in discerning which enforcement options were permissible and by whom:

*“I was familiar with law enforcement; I was not familiar with code enforcement, which is quite different… What do we do if they are selling, you know cigars that don't meet a minimum price requirement or minimum pack size? … It turns out there are a lot of options, but there's not a lot of easy options. You can do administrative citations which are less expensive but don't have much of an impact. You can do civil citations which end up being kind of a nightmare. We can suspend licenses… What needed to happen with an appeal hearing? Did we need to go to a judge? … Can we seize products? Where can we store them? Can we destroy them? Do we need a court order? Who's going to go out and do that? And so it was, it's been a really, you could probably sense it in my voice, a really frustrating situation … So, our mantra is that, you know, we're building the plane while we're flying it.”* (Local Tobacco Control Staff)

##### Municipalities differ in their available resources

Participants discussed significant differences in conducting policy implementation across municipalities; while the process was smooth in some localities, it proved challenging in others. A major contributing factor was the unequal distribution of resources, such as staffing and infrastructure. Some municipalities had sufficient staff to conduct consistent in-store education, regular inspections, and enforcement, whereas others relied on a single person, which often led to reduced enforcement efforts. Furthermore, some municipalities had an existing implementation infrastructure prior to adopting the policy, while others struggled to establish one post-adoption.

##### Insufficient knowledge of the ordinance leads to partial enforcement

Cigar pack size and pricing policies were sometimes perceived as particularly complicated when parameters differed by cigar type or had multiple policy exclusions. This led to confusion not only among retailers but also among policy implementers. Additionally, the involvement of numerous individuals or organizations across the policy adoption and implementation processes, each with varying levels of policy knowledge, resulted in implementation gaps. For instance, one participant noted that police officers were often not sufficiently familiar with the ordinance to educate retailers about their violations, which presented difficulties for tobacco control staff informing retailers about the policy:

“*Enforcement is often, you know, the police department who just kind of assigns somebody to do this. And so, they're not familiar with the ordinance as well… the retailers often aren't either… [so you] need to have a really good sense of how they are going to enforce to be able to properly educate retailers, and often these enforcement officials don't themselves have a great sense of how they're going to enforce.”* (Local Tobacco Control Staff)

##### Perceived best practices vary across implementers

When discussing their implementation approaches and comparing them to colleagues', participants described diverse perspectives on how best to handle administering fines or violations to retailers. The noted lack of standardized protocols and guidance may have led to a high degree of autonomy among individual actors, both within and across organizations.

##### Cigar use and cigar pack policies may be perceived as less of a priority

Some participants questioned the utility of extensive efforts to educate retailers on specific policy characteristics, given the confusing nature of the policy and evolving trends in youth tobacco use. For example, many participants indicated a greater focus on youth e-cigarette use as a more pressing issue, which they perceived as potentially displacing youth cigar use.

#### Barriers outside the organization

##### State laws influence local implementation

Participants reported that state laws could significantly impact local municipal policies by overriding or strengthening them. Some individuals highlighted the challenges posed by state laws that made it difficult to enforce or legitimize their local ordinances. They emphasized the critical importance of understanding the broader state and federal policy landscape and maintaining ongoing communication with state officials.

“*Products have to be in a certain pack size, or you know whatever for retail sales, but it is not illegal for them to come into [state - redacted] on a wholesale basis, and when they come into [state - redacted], they have to be stamped. They have to get a tax stamp … which kind of legalizes them in the eyes of a retailer. So, we're realizing that we need to fix some things at the state level as well.”* (Local Tobacco Control Staff)

##### Emergent events may hinder implementation

When discussing implementation efforts, many participants stated that between 2020 and 2022, during the height of the COVID-19 pandemic, their teams paused implementation efforts. Implementation, most notably inspections and enforcement, was also delayed in some municipalities. In addition to in-person visit restrictions, implementation efforts were paused broadly due to staff being shifted to other roles due to assist with pandemic relief. Participants stated that the reduced oversight likely led to the sale of illegal products.

##### A “patchwork” of policies exists across municipalities

Participants discussed the variability in policies within a state, noting that many municipalities had regulations that were discordant with those of other nearby municipalities. This led to great variation in policy parameters from locality to locality and a lack of harmony across a state or a region. Policies in neighboring communities were noted to vary for several reasons, including personal preferences regarding the minimum prices or pack sizes, fears of losing business, concerns about the ability to enforce policies, and friendly competition between communities.

## Discussion

This analysis of qualitative interviews with individuals involved in the adoption and implementation of local cigar pack size and price policies in the US offers insights into the approaches used across municipalities, alongside the facilitators and barriers encountered. Utilizing the IFASIS, a tool designed to assess factors influencing implementation and sustainment, we systematically evaluated these facilitators and barriers within their respective contexts. Our findings highlight critical areas for improvement, particularly the salience of intra-organizational barriers related to organizational readiness, implementer capacity, and delineation of responsibilities.

External factors (outside the implementing organization) represent influences that indirectly affect implementation and may not be directly modifiable by local implementers. A notable facilitator was the availability of state grant funding to address implementation costs, enabling municipalities to expand resources for retailer monitoring. On the other hand, state-level policies that appeared to conflict with local regulations emerged as a barrier, underscoring the need for awareness of the broader external policy landscape during local policy adoption.

Within the organizational context, facilitators included proactive, unofficial efforts to build capacity through self-initiated inter-agency collaborations, alongside more concerted efforts to enhance implementation via cross-agency partnerships. Cigar policy implementation often involves multiple, autonomous organizations responsible for distinct phases, ranging from policy adoption to education and enforcement. Several municipalities assigned these separate functions to different organizations. We consistently found that this structure led to discrepancies in how policies were implemented both within and across municipalities, impacting various implementation strategies and activities, from product scope to timing, methods, and personnel involved in enforcement. Consistent with prior studies [19, 20],  we found that robust interagency collaboration is essential for effective and consistent policy implementation.

Organizational barriers included broader systemic issues and challenges specific to individual policy implementers. In keeping with findings from other studies on policy implementation, a consistent lack of standardized protocols and resources was identified [19, 21]. Policy adoption often involves limited consideration for subsequent implementation, resulting in critical gaps in processes and funding once implementation activities commence. The knowledge and individual perspectives of implementers were also recognized as significant characteristics, particularly given the substantial autonomy inherent in their roles. As documented here and elsewhere [19, 22], factors such as implementers perceiving fines as excessively harsh or expressing concerns about business impacts frequently led to a greater emphasis on education over formal penalty administration. While education is crucial for implementing tobacco retail policies, it is often insufficient to ensure consistent compliance [23].

Retailers are the primary audience for interventions concerning cigar pack policies. Although some retailer characteristics cannot be altered, policymakers can mitigate implementation burdens through targeted efforts. For example, innovative educational strategies could effectively address challenges such as limited English proficiency and insufficient policy knowledge. The importance of retailer education in implementing such policies was evident in this study, aligning with findings from other research [19, 21]. While educational initiatives exist, further research into their specific components and strategies could reveal opportunities for enhancement. Similarly, this study indicated that retailers adopted new approaches to circumvent restrictions and were willing to risk policy violations. More stringent enforcement efforts may be necessary to mitigate these behaviors. The well-documented resilience of the tobacco industry in response to regulations may also be a crucial factor to consider within the broader outer context [19].

Overall, clarity was identified as a key facilitator regarding the policy itself. However, challenges related to the definition of cigars and the severity of prescribed penalties may limit overall policy effectiveness. Despite these challenges, cigar pack size and price policies were generally perceived more positively than other tobacco retailer policies. This positive perception may theoretically enhance their acceptability and subsequent implementation [24].

This study has several limitations. First, participant responses may be subject to recall bias, particularly concerning implementation activities, as some municipalities had implemented policies over a decade prior. However, participants were able to reflect on the implementation process, with no observed response variations based on time since policy adoption. Second, the interviews and analyses may have omitted important insights into policy implementation. While efforts were made to enhance applicability (e.g., clarifying concept understanding during interviews), our interpretations might still miss certain critical factors. Third, 226 representatives from municipalities with cigar pack size policies were contacted, 37 of whom participated. We are unable to determine whether there are systematic differences between participants and non-participants that may influence the findings. Finally, all participants were from four states with historically strong tobacco control programs. Therefore, the experiences, facilitators, and barriers identified in this study may not fully reflect those in states with less developed tobacco control infrastructure. However, this may emphasize the importance of addressing the identified challenges as these may be even more prominent in states without strong tobacco control programs.

## Conclusions

Local cigar policy implementation is complex, often requiring coordination across organizations and allowing significant autonomy among implementers. Ineffective implementation directly undermines policy effectiveness, limiting the potential to reduce tobacco use and its associated harms. Our conversations with individuals involved in local policy implementation suggest that the impact of policies regulating cigar pack size and price is often compromised by inconsistent interpretation and enforcement across municipalities. As a result, youth access to inexpensive cigars may persist, perpetuating tobacco-related health disparities and increasing the risk of long-term adverse health outcomes. Strengthening implementation practices is essential to realizing the full public health benefit of these policies, particularly in communities disproportionately burdened by tobacco use. Moreover, variability in implementation should be systematically accounted for when evaluating policy effectiveness. Applying an implementation science framework to identify and address facilitators and barriers can support municipalities in developing proactive strategies to enhance policy success and reduce tobacco-related harm.

## Supplementary Information

Additional file 1: COREQ Checklist (.pdf). Includes references to where specific features noted in the qualitative reporting checklist are located in the manuscript.

Additional file 2: Interview guide (.pdf). Includes the semi-structured interview guide used to conduct the qualitative interviews.

Additional file 3: Exemplar quotes (.pdf). Includes exemplar quotes for all themes.

## Data Availability

The datasets used and/or analysed during the current study are available from the corresponding author on reasonable request.

## Abbreviations

US: United States
IFASIS: Inventory of Factors Assessing Successful Implementation and Sustainment
COREQ: Consolidated Criteria for Reporting Qualitative Studies
