# Supplementary material for: “We’re building the plane while we’re flying it”: perspectives on local cigar policy implementation from qualitative interviews with key personnel
Source: Implement Sci Commun. 2026 Jan 16;7:30. doi: 10.1186/s43058-026-00864-8 (PMC12892670; doi:10.1186/s43058-026-00864-8)
Supplement: Supplementary file 1 — Additional file 1: COREQ Checklist (.pdf). Includes references to where specific features noted in the qualitative reporting checklist are located in the manuscript. [file 43058_2026_864_MOESM1_ESM.pdf]

Additional File 1: Consolidated Criteria for Reporting Qualitative Studies (COREQ)

	Item	Guide questions/description	Page
**Domain 1: Research Team and Reflexivity**			
Personal Characteristics			
1.	Interviewer/facilitator	Which author/s conducted the interview or focus group?	7
2.	Credentials	What were the researcher's credentials? *E.g. PhD, MD*	7
3.	Occupation	What was their occupation at the time of the study?	7
4.	Gender	Was the researcher male or female?	7
5.	Experience and training	What experience or training did the researcher have?	7
Relationship with participants			
6.	Relationship established	Was a relationship established prior to study commencement?	7
7.	Participant knowledge of the interviewer	What did the participants know about the researcher? e.*g. personal goals, reasons for doing the research*	7
8.	Interviewer characteristics	What characteristics were reported about the interviewer/facilitator? e.g. *Bias, assumptions, reasons and interests in the research topic*	7
**Domain 2: Study Design**			
Theoretical framework			
9.	Methodological orientation and Theory	What methodological orientation was stated to underpin the study? *e.g. grounded theory, discourse analysis, ethnography, phenomenology, content analysis*	6
Participant selection			
10.	Sampling	How were participants selected? *e.g. purposive, convenience, consecutive, snowball*	7
11.	Method of approach	How were participants approached? e.*g. face-to-face, telephone, mail, email*	7
12.	Sample size	How many participants were in the study?	7
13.	Non-participation	How many people refused to participate or dropped out? Reasons?	7
Setting			
14.	Setting of data collection	Where was the data collected? e.*g. home, clinic, workplace*	7
15.	Presence of non-participants	Was anyone else present besides the participants and researchers?	7
16.	Description of sample	What are the important characteristics of the sample? *e.g. demographic data, date*	6, 7
Data collection			
17.	Interview guide	Were questions, prompts, guides provided by the authors? Was it pilot tested?	7
18.	Repeat interviews	Were repeat interviews carried out? If yes, how many?	N/A
19.	Audio/visual recording	Did the research use audio or visual recording to collect the data?	7
20.	Field notes	Were field notes made during and/or after the interview or focus group?	7
21.	Duration	What was the duration of the interviews or focus group?	7
22.	Data saturation	Was data saturation discussed?	N/A

	Item	Guide questions/description	Page
23.	Transcripts returned	Were transcripts returned to participants for comment and/or correction?	N/A
**Domain 3: Analysis and Findings**			
Data analysis			
24.	Number of data coders	How many data coders coded the data?	7-8
25.	Description of the coding tree	Did authors provide a description of the coding tree?	N/A
26.	Derivation of themes	Were themes identified in advance or derived from the data?	7-8
27.	Software	What software, if applicable, was used to manage the data?	7
28.	Participant checking	Did participants provide feedback on the findings?	N/A
Reporting			
29.	Quotations presented	Were participant quotations presented to illustrate the themes / findings? Was each quotation identified? e.*g. participant number*	9-18
30.	Data and findings consistent	Was there consistency between the data presented and the findings?	9-21
31.	Clarity of major themes	Were major themes clearly presented in the findings?	8-21
32.	Clarity of minor themes	Is there a description of diverse cases or discussion of minor themes?	8-22
