# Supplementary material for: “We’re building the plane while we’re flying it”: perspectives on local cigar policy implementation from qualitative interviews with key personnel
Source: Implement Sci Commun. 2026 Jan 16;7:30. doi: 10.1186/s43058-026-00864-8 (PMC12892670; doi:10.1186/s43058-026-00864-8)
Supplement: Supplementary file 2 — Additional file 2: Interview guide (.pdf). Includes the semi-structured interview guide used to conduct the qualitative interviews. [file 43058_2026_864_MOESM2_ESM.pdf]

Additional File 2: Cigar Pack Policy Interview Guide

A. Pre-Interview Process (3-5 minutes)

1. Welcome the participant and introduce yourself and others on call.
2. Give brief information about the project:
   This is part of an FDA/NIH-funded study looking at the impact of cigar pack size and price policies. We want to learn about how cigar pack policies were adopted, implemented, and enforced to inform FDA on future regulations.
3. Describe the format of the interview:
   Today we will talk for 45-60 minutes. I'll ask a series of open questions to explore your perspectives. I have an interview guide I'll be following to keep us on track, but the main goal is to learn from you about what happened in your community with these policies.
4. ASK: Do you have any questions?
5. Review consent, explain confidentiality (e.g., anonymity, data protection)
   - READ: *I understand you've received a copy of the informed consent. There are a few things I want to highlight – first, we will do what we can to maintain confidentiality. For example, we will only want to use first names during our discussion today. We will also only report aggregated information – we won't share information about a specific city or county.*
   - ASK: What questions do you have about the study or procedures?
   - ASK: Do you give your permission for the interview to be audio recorded?
     - Ensure verbal consent is received.
6. Start recording

B. Warm Up (5 minutes)

- Let's start by having you share with us about your position, including how long you've been with the organization and what you do in your current role.
  - Potential probes:
    - How does your role or organization relate to tobacco control?
- What's the tobacco/cigar culture like in [community interview is about]?
  - Probes if not mentioned:
    - What are the norms around smoking? Are the norms different for adults vs youth?
    - Is reducing use a priority?
    - What does retail access look like? How does it compare to nearby communities?

C. Cigar Pack Ordinances (40-50 minutes)

- As you know, today we are talking about cigar pack size and pricing ordinances. These are often adopted as part of a larger tobacco control package, with perhaps restrictions on flavors or age of sale, but today we specifically want to discuss and learn more about the restrictions on cigar pack size and price. So, when responding, please try to tailor your response specific to the cigar pack size or minimum price component when possible. [Note: they should be aware, but if they appear unclear, consider providing additional detail]
  How familiar are you with cigar pack size or cigar minimum price ordinances?
  In what capacity?
  - Potential probes if not mentioned:
    - Did you work here when the ordinance was initially adopted and implemented?
    - Has the ordinance changed during the time you have been working here?

- [If involved in/aware of policy adoption process] What do you recall about the adoption process?
  - How was the cigar pack size or price conversation initiated?

        - Do you recall who initiated the conversation or when?
        - Any tobacco advocacy groups?
    - Were there discussions in board meetings?
        - What were those like? Who attended? Anything beyond standard procedures?
    - What talking points are used about the policies?
        - (maybe youth prevention or increasing revenue for retailers; could also capture whether these are price or pack size policies)
    - What challenges were there in getting the ordinance adopted?
        - What changes were made to the ordinance prior to adoption?
        - Who supported /opposed the ordinances?
        - Why were these specific bans/restrictions implemented? [note anything unique about specific ordinance – will be in guide]
        - Were there any discussions about equity?
    - [for those working in/across multiple localities] What differences were there across localities regarding the ordinance adoption process?
        - Or were there changes over time?

- [If involved in/aware of initial implementation process] What can you tell me about how the ordinance was implemented?
    - What retailer education efforts such as signage or compliance checks were implemented?
        - Do those efforts work?
    - Were these efforts specific to cigar pack size and price or are efforts broadly across tobacco regulations?
    - Do you recall if there were any news or media related to the ordinance?
    - [if ordinance differentiates between cigar type based on weight] – I noticed that the ordinance differentiates between different types of cigars based on their weight – how is this monitored or assessed?

BRING THE CONVO BACK TO CIGAR PACK SIZE AND PRICE AS NEEDED

- The ordinance has been in place XX months/years. What have you observed since then?
    - Potential probes:
        - Any pushback from community members or businesses?
        - Tobacco industry tactics?
        - What works well and why?
        - What challenges are there?
        - What support needed?

- What are the current enforcement efforts?
    - Are there compliance checks? Who, frequency? Is there funding for it?
    - Have there been violations? How many, who, more/less than other tobacco regulations (eg flavors, age of sale)?

- What changes have you noticed in the retail environment?
    - What changes with regard to pack size (single, 2-pack availability)
    - what changes with regard to price? more expensive? are less expensive products still sold

- How would you describe the impact of these ordinances overall?
    - How have they impacted tobacco use?
    - Any unanticipated/surprising features?

    - How would you compare the impact of this policy compared to other aspects of the tobacco regulations, like age of sale, flavors?

- [share relevant study findings based on location] What are your thoughts on these findings? Does this align with what you've seen? Any thoughts on why we did/didn't find…
- We've seen a lot of variety in the pack size and prices these ordinances are set at. For example [compare to ordinances in other cities/states], do you think the impact would be different if your locality implemented one of these regulations?
- [if time allows or if it presents itself] Can you think of other factors that might be influencing the policies
    - (maybe retailer knowledge, maybe cross-border sales, unlicensed retailers – especially related to cannabis)

- When issues arise – whether in the adoption, implementation, or enforcement phases – who do you reach out to for guidance?
- What advice or lessons learned do you have for other localities adopting these policies?
- Are there any data or resources that would be helpful for you to have had when adopting the policies, implementing, or in ongoing enforcement?

D. Closing (2 minutes)

- What else would you like to discuss on the topic?
- Once complete, turn off recording

E. Post-Interview Process (5 minutes)

1. Thank participant for their valuable contributions to the project and let them know we will send $50 Amazon gift card by email.
2. Ask if there is anyone else who would be good for us to talk to about cigar pack policies. If they say yes, get their info.
3. Let participant know that we plan to send summary info with the next year. Team is also happy to present to group on pack size policies.
4. Complete the field notes.
