# Supplementary material for: “We’re building the plane while we’re flying it”: perspectives on local cigar policy implementation from qualitative interviews with key personnel
Source: Implement Sci Commun. 2026 Jan 16;7:30. doi: 10.1186/s43058-026-00864-8 (PMC12892670; doi:10.1186/s43058-026-00864-8)
Supplement: Supplementary file 3 — Additional file 3: Exemplar quotes (.pdf). Includes exemplar quotes for all themes. [file 43058_2026_864_MOESM3_ESM.pdf]

Additional File 3: Exemplar quotes from qualitative analysis.

**Local Policy Implementation Components and Process**	
Grace Period between Adoption and Enforcement	"You'd pick an effective date for the policy to go into effect, and we would always pick a date that was usually about six weeks or more out, so that our programs could do the educational piece that they needed to do for the retailers." (Statewide Technical Assistance Provider)
In-Person Educational Visits	"I visited every single store in person with a copy of that letter from the Board because we do know that, you know, store owners are busy. Especially small stores are doing a million things. They don't always open their mail. So it was a personal visit: 'Have you seen this letter? You need to know this, and this is what you need to do.'" (Local Tobacco Control Staff)<br><br>"In other places they weren't complying with it and they didn't really understand how to comply because they weren't very well educated on it. So, we were trying to increase their education around that kind of one-on-one." (Local Tobacco Control Staff)
Educational Materials	"So we kind of put together educational materials, you know, with flyers and pictures of the products… when we talked about a 10-pack size, what that meant, what the exemptions to that were because that was really confusing." (Local Tobacco Control Staff)
**Facilitators and Barriers to Implementation, aligned with the IFASIS Tool**	
Facilitators Outside the Organization	
Grant funding was sometimes available to address costs.	"So [redacted] State Department of Justice has had tobacco-related enforcement grants over the past… since, like, 2018. And so there have been some local departments, local sheriff departments or police departments, that have really used that money well to kind of figure out enforcement." (Local Tobacco Control Staff)
Facilitators Within the Organization	
Implementers engaged in unofficial efforts to build capacity.	"In some of our jurisdictions that passed tobacco retail licensing ordinances, there has not been really strong enforcement. And, so, we have developed a [redacted location] Tobacco Retail Enforcement Network to bring enforcement officials, tobacco retail enforcement officials together to try to, you know, just kind of build their capacity and their understanding about the various policies and enforcement best practices and strategies." (Local Tobacco Control Staff)
Implementation is a collaborative effort across organizations.	"We partner with our police department and sort of use their expertise in how to approach the enforcement side of it. So the trainings were a partnership of code enforcement and the Police Department." (Community Development Director)
Facilitators related to Cigar Pack Policies	

Clear and enforceable ordinances improve implementation.	“Being able to kind of think ahead to how it will be implemented and who is going to be doing that and how much it will cost is always a good idea…we don't want to adopt the policy that we can't enforce.” (Local Tobacco Control Staff)
Less retailer pushback toward cigar pack size and price policies.	“Our strategy is we want to give the retailer a heads up that we're putting the price of tobacco up now, use that extra money so that you can buy a freezer or produce case and transition over to something else, because down the road we are going to ban the sale of tobacco products… use this to transition to another product that's going to allow you to stay whole as a business.” (Local Tobacco Control Staff)
Barriers Outside the Organization	
State laws influence local implementation.	“Products have to be in a certain pack size, or you know whatever for retail sales, but it is not illegal for them to come into [state - redacted] on a wholesale basis, and when they come into [state - redacted], they have to be stamped. They have to get a tax stamp … which kind of legalizes them in the eyes of a retailer. So, we're realizing that we need to fix some things at the state level as well.” (Local Tobacco Control Staff)
Emergent events may hinder implementation.	[referring to COVID-19 impacts] “I think maybe some store owners didn't think we were going to be doing it, assumed we wouldn't be doing it. We're still masked at that point.” (Local Tobacco Control Staff)
“Patchwork” of policies across municipalities.	“There began to be some variety [in policy parameters], and I think it was just a bit of friendly competition…the next community to adopt upped the minimum price of the single cigar to $2.60. They just added $0.50 to the minimum price, and there wasn't like a lot of kind of math, economics behind it. They're just like we want to be $0.50 more expensive…we want to be that much better than this first community.” (Local Tobacco Control Staff)
Barriers Within the Organization	
Lack of protocols for inspections and enforcement.	“We have trainings on enforcement, but none of them have actually been too strong on how you actually conduct yourself doing these inspections or compliance checks. And how you actually protect yourself from the dangers of going to a place; like people have guns behind the counter sometimes” (Local Tobacco Control Staff)<br><br>“I was familiar with law enforcement; I was not familiar with code enforcement, which is quite different… What do we do if they are selling, you know cigars that don't meet a minimum price requirement or minimum pack size? … It turns out there are a lot of options, but there's not a lot of easy options. You can do administrative citations which are less expensive but don't have much of an impact. You can do civil citations which end up being kind of a nightmare. We can suspend licenses… What needed to happen with an appeal hearing? Did we need to go to a judge? … Can we seize products? Where can we store them? Can we destroy them? Do we need a court order? Who's going to go out and do

	that? And so it was, it's been a really, you could probably sense it in my voice, a really frustrating situation … So, our mantra is that, you know, we're building the plane while we're flying it." (Local Tobacco Control Staff)
Differences in resources across municipalities.	"When it comes to compliance, it really depends on the program. Well, it depends, it depends on the enforcement. Basically, if you're if you have a municipality like [redacted – large city], they have incredible compliance rates because they have a director and they have a staff that are out all the time … versus if you go to [redacted – smaller city], which is an urban city in [redacted state], we have one director and no staff, and really no support in the city, and you can get anything you want. So, it really depends." (Statewide Technical Assistance Provider)
Insufficient knowledge of ordinance leads to partial enforcement. Cigar	"Enforcement is often, you know, the police department who just kind of assigns somebody to do this. And so, they're not familiar with the ordinance as well… the retailers often aren't either… [so you] need to have a really good sense of how they are going to enforce to be able to properly educate retailers, and often these enforcement officials don't themselves have a great sense of how they're going to enforce and they don't really want to tell you all about how they can enforce or not enforce." (Local Tobacco Control Staff)
Perceived best practices vary across implementers.	"I think my way is the best way… I've talked to some of these folks and yeah, and some of them are a lot stricter. It's like they go in there, and boom, $500 fine… I've found, especially with restaurants and other things, if you beat people over the head with a hammer right away, you get less compliance than if you try to help them. And I almost had no choice... I had to do observational educational inspections and put everything into an observation. And not hit them with any true violations the first time out or we would have no tobacco stores in [redacted city name], cause I would have shut everyone down. I literally would have shut everyone down the first inspection. And that is not gonna fly in [city name]. It's just you politically cannot do that." (Local Public Health Staff)
Cigar use and cigar pack policies may be perceived as less of a priority.	"It's not worth the work to raise that price because retailers were so confused at the beginning [about why the cigar policy was being implemented]... If we saw a lot of youth use still going on, which I just don't see that as an issue. And so many retailers have gotten rid of the single [cigar] anyway." (Local Tobacco Control Staff)
Barriers among Retailers	
Retailers creatively evade restrictions.	"So because $2.50 is a lot to pay for a single [cigar], and you want to make that 5 pack cheaper [per stick] than the single… there were a lot of two packs around… and they taped or elasticized two two-packs. So that now it's a made-up four-pack for $5, which made it $1.25 per cigarillo." (Local Tobacco Control Staff)
Limited English proficiency among retailers.	"Some of the things that we constantly come up against is, you know, the diversity in the stores that we visit. You know, some of the language barriers that we come across, and you're trying to get these rules and regulations and policies in place and really getting

	them to understand what they mean and what the outcome will be if there are violations." (Local Tobacco Control Staff)
Insufficient product education	"I think with cigars, one of the things that we found is that retailers were really confused about what was the cigar, what was the cigarillo, because it doesn't matter if the manufacturer labeled them as cigars, if they don't meet the legal definition, they're not cigars. So it would have been helpful to have had better explanation, and I think pictures of products that they could have and they couldn't have. It was hard to explain it to them. We give them, you know, all this information, they didn't want to read it and they, even when they would read it, they didn't understand. So having real clear, I think, pictures of acceptable products and not acceptable products." (Local Tobacco Control Staff)
Retailers willing to risk policy violations	"I would say the biggest kind of unintended consequences that people are just gonna have, like, a box of singles and then for their regular customers, they're just gonna be selling those. You know, it's kind of the obvious thing to do for them, especially when they're only getting visited maybe once a year, and if that's their only violation, they're not going to have a stiff penalty for that. So that's probably, you know, it's safe to say that that's happening." (Local Tobacco Control Staff)
Barriers related to Cigar Pack Policies	
Penalties may not be sufficient to discourage retailers from selling restricted products.	"These things sell, and they're trying to make money. As simple as that. I think it all boils down to the mighty dollar. And why I'm getting resistance from some of these people and doing these backdoor type of things, especially as more and more people comply. Now they're more and more and more in demand. So they're making more and more and more money, you know, so they're taking that risk. And they don't think that the penalties are all that much. They're not going to go to jail." (Local Public Health Staff)
Challenges with weight-based cigar definitions.	"Whatever company makes Black and Mild reached out to them and said 'hey our product is not a little cigar, it exceeds, you know, the cutoff for that product. It's actually a large cigar by your ordinance definitions'. And then we've had to send out communication to all retailers and say… this is now considered a large cigar, which for them for something like Black and Mild is just preposterous to think that they're charging that much money for that. And so that then becomes another thing for them to figure out: 'Do I carry this product?' 'How do I communicate to customers that this is now a large cigar and what was $0.99 is now $8?" (Local Tobacco Control Staff)
